# Lipid Profile in Olympic Athletes: Proposal for a “Lipid Athlete Score” as a Clinical Tool to Identify High-Risk Athletes

**DOI:** 10.3390/jcm12237449

**Published:** 2023-11-30

**Authors:** Giuseppe Di Gioia, Lorenzo Buzzelli, Viviana Maestrini, Antonio Nenna, Sara Monosilio, Maria Rosaria Squeo, Erika Lemme, Antonio Pelliccia

**Affiliations:** 1 Department of Cardiology, Institute of Sport Medicine and Science, National Italian Olympic Committee, Largo Piero Gabrielli, 1, 00197 Rome, Italy; vivianamaestrini@gmail.com (V.M.); sara.monosilio@gmail.com (S.M.); mariarosaria.squeo@coni.it (M.R.S.); erikalemme@msn.com (E.L.); ant.pelliccia@gmail.com (A.P.); 2 Department of Cardiovascular Sciences, Fondazione Policlinico Universitario Campus Bio-Medico, Via Alvaro del Portillo, 200, 00128 Rome, Italy; lorenzo.buzzelli@unicampus.it; 3 Department of Movement, Human and Health Sciences, University of Rome “Foro Italico”, Piazza Lauro De Bosis, 15, 00135 Rome, Italy; 4 Department of Clinical, Internal, Anesthesiologic and Cardiovascular Sciences, Sapienza University of Rome, Piazzale Aldo Moro, 5, 00185 Rome, Italy; 5 Department of Heart Surgery, Fondazione Policlinico Universitario Campus Bio-Medico, Via Alvaro del Portillo, 200, 00128 Rome, Italy; a.nenna@policlinicocampus.it

**Keywords:** Olympic athletes, endurance exercise, strength training, concurrent exercise, lipid athlete score, dyslipidemia

## Abstract

Dyslipidemia is a major risk factor for atherosclerosis. Identification of dyslipidemia in athletes has raised interest in establishing preventive strategies and reducing cardiovascular (CV) events. Nowadays, targets or “scores” for athletes are undefined. The aim of our study was to create a “Lipid Athlete Score” based on lipid parameters and derive score indexes to identify high-risk athletes. We retrospectively enrolled 957 Olympic athletes practicing different sporting disciplines (power, skills, endurance, and mixed), analyzing their CV profiles and anthropometrics; 55.4% were male, the mean age was 27.1 ± 5 years, and the mean body mass index (BMI) was 23.1 ± 3.2 kg/m^2^. Three hundred and forty-three athletes (35.8%) were dyslipidemic (LDL ≥ 115 mg/dL or LDL/HDL ≥ 1.90). Multivariate analysis revealed the following: male *p* = 0.001, OR 1.88 [0.41–2.51], familiarity for dyslipidemia *p* = 0.001, OR 2.82 [1.72–4.59], BMI ≥ 30 kg/m^2^ *p* = 0.001, OR 2.53 [1.46–4.38], and fat mass *p* = 0.001, OR 2.29 [1.38–3.80] were significant. Endurance athletes presented the lowest CV risk. We proposed a lipid athlete score including major (LDL ≥ 115 mg/dL and LDL/HDL ≥ 1.90) and minor criteria (male, BMI > 30 kg/m^2^ or fat mass >22% for males and 32% for females, familiarity for dyslipidemia, and conventional CV risk factors). Twelve athletes (1.2%) were at high risk, 150 athletes (15.7%) at medium risk, 171 athletes (17.9%) at low risk, and 624 (65.2%) were at no risk. Dyslipidemia is very common in elite athletes. We have defined a specific lipid athlete score based on lipid parameters and derived score indexes for the stratification of risk. In accordance with this tool, a substantial proportion of athletes (16.9%) were at medium-to-high risk and need early preventive strategies to improve their lipid profiles and reduce the future development of atherosclerotic CV diseases.

## 1. Introduction

Cardiovascular (CV) diseases, as a consequence of atherosclerotic complicated vascular damage, are the main determinants of global mortality and morbidity, leading international scientific societies to encourage proper preventive strategies. In this assumption, assessment of CV risk scores is nowadays the cornerstone of prevention [1].

Regular physical exercise plays a key role in this context, as it tempers the prevalence and magnitude of CV risk factors and is consequently associated with lower rates of CV diseases.

Therefore, Olympic athletes are supposed to have a protective and favorable CV risk profile, as a result of their usual young age and regular training programs, and they are intuitively considered a healthy lifestyle paradigm. However, this assumption has not been supported by robust and extensive data. In addition, previous observational studies have found a high prevalence of dyslipidemia in professional athletes. In a cohort of 1058 elite athletes, dyslipidemia, defined as a low-density lipoprotein (LDL) level > 115 mg/dL, was the most common CV risk factor (32%) [2]. Other previous studies consistently found a 20% incidence of dyslipidemia in professional skiers and 30% in professional cyclists [3].

Therefore, the identification of dyslipidemia in a young, athletic population is not a trivial issue and has recently raised novel interest [4] in establishing a lifestyle modification program (or an early treatment) to avoid the future development or progression of subclinical atherosclerosis. 

The definition of the best target to use in athletes is also complicated by the unsuitability of both SCORE and pooled cohort equations in athletes, since the calculation of CV risk by them can only be applied to individuals over 40 years old and does not take into account physical fitness [5,6].

In the current clinical practice, LDL cholesterol is still considered the prime index of CV risk [7,8]; however, several lipoprotein ratios or “atherogenic indices”, like the LDL/high-density lipoprotein (HDL) ratio, total cholesterol (TC)/HDL cholesterol ratio, or non-HDL cholesterol levels, have been proposed as indexes to enhance the predictive ability of the lipid profile [9]. Their use as predictors of CV risk comes from epidemiological studies that have validated these ratios as better predictors than simple lipid parameters [10,11]. Nevertheless, the current American and European guidelines still consider LDL cholesterol as the main target of plasma lipid control [7,8]. At present, specific targets or “scores” for athletes, such as LDL, lipid ratios, or others, are undefined.

In the present study, we analyzed the lipid profile in a large cohort of Olympic athletes of both sexes and different sporting disciplines. We aimed to evaluate the prevalence of dyslipidemia in elite athletes, the possible correlation with clinical and anthropometrical parameters, and the influence of different types of sporting disciplines. We therefore set out to define a “Lipid Athlete Score” based on lipid parameters and derive score indexes to identify subjects who can be defined as high risk and need early correction of their CV risk profile.

## 2. Materials and Methods

The Institute of Sport Medicine and Science in Rome is the division of the Italian Olympic Committee responsible for the medical evaluation of the elite athletes selected for participation in the Olympic Games.

In our study, we included a large cohort of 958 Olympic athletes evaluated in a 10-year period (from the 2012 London Summer Games to the 2022 Beijing Winter Olympic Games). All were world-level competitors, and a substantial proportion (30%) had participated in more than one Olympic Game. Athletes taking either statins, ezetimibe, or nutraceutical supplements with anti-lipidic actions were excluded from the study; the final cohort comprised a total of 957 athletes. Pre-participation screening in Olympic athletes included complete blood tests, rest ECGs, exercise stress tests, transthoracic echocardiograms, and multidisciplinary clinical evaluations.

Athletes were engaged in a wide spectrum of sport disciplines, classified into four groups, as previously described [2]:

(1) Power (strength disciplines): weightlifting, Greco-Roman wrestling, judo, javelin, shot-putting, bobsleigh, skeleton, snowboard, swimming (<800 mt), alpine skiing, athletics (sprinting, shot putting, and discus), and luge.
(2) Skills (technical disciplines): archery, equestrian, golf, shooting, figure skating, sailing, curling, diving, and equestrian sports.
(3) Endurance (primarily dynamic components): cycling, rowing, canoeing, triathlon, long-distance running, long-distance swimming (>800 mt), cross-country skiing, pentathlon, biathlon, speed-skating, and Nordic combined.
(4) Mixed disciplines (alternate dynamic and strength components): soccer, volleyball, basketball, tennis, fencing, water polo, rhythmic gymnastics, taekwondo, badminton, beach volley, and softball.

Blood pressure was recorded in the sitting position before exercise testing, as recommended [12]. Body height and weight were obtained in each subject, and the body mass index (BMI) was calculated as weight (kg)/height (m)^2^. Body surface area (BSA) was derived from the Mosteller formula [13].

Body composition and fat mass percentage measurements were made with Bioelectric Impedance Analysis (BIA 101 Quantum, Akern, Italy) with a constant sinusoidal current at an intensity of 50 kHz and 400 μA, respectively. Food habits and history were collected, and elements of diet composition (fats, proteins, and carbohydrates) were calculated.

CV risk factors were defined as follows:

Family history for atherosclerosis (ATH): fatal or non-fatal CV events or/and established diagnosis of CV disease in first-degree male relatives before 55 years of age, or female relatives before 65 years of age [12], or evidence of carotid/peripheral atherosclerotic disease in first-degree relatives;
Family history for dyslipidemia: first-degree relative in treatment (pharmacological or nutraceutical);
Cigarette smoking: defined as regular smokers of at least one cigarette per day;
Overweight: in order to avoid misclassifying subjects with increased muscular mass and high BMI as overweight, our definition of “overweight” was a waist circumference of >94 cm for men or >80 cm for women [12], or a BMI over 30;
Fat mass: normal values were identified as 10% to 22% body fat in males and 20% to 32% in females [14];
Hypertension: systolic blood pressure ≥ 140mm Hg and/or diastolic ≥ 90 mm Hg, or subjects on pharmacological antihypertensive therapy [12];
Diabetes: diagnosis was made in case of fasting glucose ≥126 mg/dL or current treatment with insulin or antidiabetic drugs [15];
Dyslipidemia: was defined as LDL ≥ 115 mg/dL [15,16], HDL < 40 mg/dL for males or HDL < 50 mg/dL for females, and a LDL/HDL ratio > 2.78 [17];
Hypertriglyceridemia: triglycerides (TG) values superior to 150 mg/dL [16];
Alcohol intake: we used a binary classification: “none” if the athlete did not drink and “user” if assumed at least one glass of wine/beer or super-alcoholic for week.

Blood samples were collected early in the morning and after at least 10 h of fasting and were analyzed on the same day. All blood tests (from 2012 to 2022) were collected and analyzed in the same laboratory.

In addition, clinical evaluations were performed and medical histories were collected to rule out secondary causes of dyslipidemia [18]. Specifically, in 51 athletes (5.3%), a thyroid disorder was present; however, their lipid profile and lipid ratio results were similar to those without thyroid disease (TC, *p* = 0.911; LDL, *p* = 0.57; HDL, 0,64; TG, *p* = 0.642; and LDL/HDL, *p* = 0.469). Thus, these athletes were included in the study.

The study design of the present investigation was evaluated and approved by the Review Board of the Institute of Medicine and Sports Science (date of approval: 23 February 2023; IRB approval code: cni2302202). All athletes included in this study were fully informed of the types and nature of the evaluation and signed the consent form, according to Italian Law and Institute policy. All clinical data collected from the study population are stored in an institutional database. The work described was performed in accordance with The Code of Ethics of the World Medical Association (Declaration of Helsinki).

## 3. Statistical Analysis

Categorical variables were expressed as frequencies, and percentages were compared using the Fisher’s exact test or Chi-square test, as appropriate. Normality criteria were checked for any continuous variable, which were presented as the mean ± standard deviation and compared using the Student’s *t*-test for independent data if normally distributed; otherwise, median and interquartile range were shown, and the data were compared with the Mann–Whitney test or Kruskal–Wallis test (with Dunn’s test for post-hoc comparisons). Cut-off analysis was performed with the “dtroc” command, considering the maximum efficiency criteria. Variables were included in the multivariate logistic regression models using a stepwise approach in the case of univariate *p*-values less than 0.20. Models were compared using the partial likelihood ratio test, and post-estimation commands (goodness-of-fit was evaluated using the Hosmer–Lemeshow test and area under the ROC curve (AUC)) were performed to evaluate overall model fitness. A two-tailed *p*-value less than 0.05 was considered statistically significant. Statistical analysis was performed with STATA Statistics for Windows (SE, version 17). Considering the retrospective nature of this article, no “a priori” sample size calculation was performed.

## 4. Results

Nine hundred and fifty-seven Olympic athletes have been studied. Five hundred and thirty (55.4%) were male; the mean age was 27.1 ± 5 years old (ranging from 15 to 47 years old); the mean BMI was 23.1 ± 3.2 kg/m^2^, and the mean fat mass was 15.8 ± 6.6%; most of them were Caucasian (923, 96.4%). Three hundred and eleven athletes (32.5%) practiced power disciplines, 122 (12.8%) skills, 264 (27.6%) endurance, and 260 (27.1%) mixed sports. As CV risk factors, two athletes had type 1 diabetes, one athlete presented type 2 diabetes, and four athletes (0.4%) had hypertension taking chronic pharmacological therapy. Seventy-seven athletes (8%) were active smokers, 225 (23.5%) had a family history for ATH, and 78 (8.1%) had a family history for dyslipidemia. Finally, 25 athletes (2.6%) were reported as overweight (with a BMI > 30 kg/m^2^).

## 5. Lipid Profile Analysis

The results describing the CV risk and lipid profile in athletes according to the sport they practiced are reported in Table 1. Endurance athletes presented the lowest global CV risk (fewer smokers, lower body weight, BMI, and fat mass) compared to other types of sports, despite their higher kcal daily intake. Furthermore, a significant increase in HDL cholesterol was detected (71.6 ± 16.3 mg/dL in endurance vs. 62.8 ±14.9 mg/dL in power, 61 ± 16.5 mg/dL in skills, and 66.4 ± 14.6 mg/dL in mixed sports; *p* = 0.001), while there were no differences in TC (180.3 ± 31.4 mg/dL for endurance, 175.6 ± 33.1 mg/dL for power, 175.3 ± 34.7 mg/dL for skills, and 182.5 ± 32.3 mg/dL for mixed; *p* = 0.075) and LDL (95.1 ± 26.9 mg/dL for endurance, 97.8 ± 27.1 mg/dL for power, 97.7 ± 29.2 mg/dL for skills, and 102 ± 28.1 mg/dL for mixed sports; *p* = 0.100). Instead, higher levels of TG were observed in athletes practicing skill disciplines (85.1 ± 56.9 mg/dL vs. 68.6 ± 28.7 mg/dL for endurance, 75.7 ± 40.5 mg/dL for power, and 73.8 ± 36.4 mg/dL for mixed sports; *p* = 0.009).

Significant gender differences in lipid profiles were observed (Table 2).

Four hundred and twenty-seven athletes (44.6) were female, with a mean age of 26.4 ± 4.8 vs. 27.6 ± 5 of male athletes (*p* = 0.005). Women showed a lower CV risk profile, with a lower body weight (62.7 ± 10 vs. 81.6 ± 13.5 kg in males; *p* = 0.001), lower BMI (21.7 ± 2.6 vs. 24.2 ± 3.2 kg/m^2^ in males, *p* = 0.001), and lower waist circumference. Moreover, they had a better lipid profile with higher HDL levels (72.4 ± 14.9 mg/dL vs. 60.7 ± 14.8 mg/dL of males, *p* = 0.001), lower LDL levels (95 ± 25.1 mg/dL vs. 100.8 ± 29.4 mg/dL in males, *p* = 0.002), and LDL/HDL ratio (1.36 ± 0.5 vs. 1.76 ± 0.7 in male, *p* = 0.001).

The analysis of the body weight and fat mass of the athletes (Table 3) showed a direct correlation between the increase in both variables and the increase in TC, LDL, and TG, with a progressive reduction in HDL cholesterol. The LDL/HDL ratio was statistically connected to the increase in body weight and fat mass. Of note, overweight and increased fat mass were not present in endurance athletes.

## 6. Identification of a Cut-Off for Dyslipidemia

We then used the proposed definition of dyslipidemia (LDL ≥ 115 mg/dL, HDL < 40 mg/dL for males or HDL < 50 mg/dL for females or a LDL/HDL ratio > 2.78) [17], and we found that 287/957 (30%) athletes fitted in at least one of these three definitions. Namely, 245 (25.6%) athletes had LDL ≥ 115 mg/dL; 53 (5.5%) had HDL < 40 mg/dL for males or < 50 mg/dL for females, and 52 (5.4%) had a LDL/HDL ratio > 2.78.

We evaluated the so-identified “dyslipidemia” categories as dependent variables, and from the cut-off analysis we confirmed the actual value of LDL ≥ 115 mg/dL as the best cut-off (AUC = 0.93; [95% CI 0.91–0.95]); sensibility = 87.2% (95% CI 82.8–90.6%); specificity = 100% (95% CI 0.026–0.841); positive predicted value (PPV) + 100%; and negative predictive value (NPV) + 94.9%. The same analysis showed poorer results for HDL cut-off to identify dyslipidemic athletes (male: AUC = 0.37 [95% CI 0.33–0.41]; female: AUC = 0.44 [95% CI 0.40–0.49]). Finally, with regard to the LDL/HDL ratio, the optimal cut-off resulted 1.90 (maximal efficacy criteria); Se 66.9 [95% CI 61.2–72.1]; Sp 91.6 [95% CI 89.3–93.5]; and PPV +77%; NPV 86.8%.

LDL/HDL ≥ 1.90 was present in 188 athletes, 19.6% of the overall population, and 65.5% of the dyslipidemic athletes.

So, by removing the HDL cut-off from the definition of “dyslipidemia” and only considering the LDL cut-off ≥ 115 mg/dL and the “new” LDL/HDL ratio ≥ 1.90, we identified 56 additional athletes that should be defined as dyslipidemic, with a total of 343/957 (35.8%).

## 7. Univariate and Multivariate Analyses

Starting from the new definition of “dyslipidemia” in athletes (LDL ≥ 115 mg/dL or LDL/HDL ratio ≥ 1.90), we performed univariate and multivariate logistic regression analyses.

At univariate analysis, the following parameters were independently related to dyslipidemia: male sex *p* = 0.001 OR 2.21 [1.68–2.92], familiarity for dyslipidemia *p* = 0.001 OR 2.65 [1.66–4.25], age ≥ 28 years old *p* = 0.001 OR 1.04 [1.01–1.07], BMI ≥ 30 kg/m^2^ *p* = 0.001 OR 1.17 [1.11–1.22], fat mass in males > 22% *p* = 0.001 OR 1.10 [1.06–1.15], and fat mass in females > 32% *p* = 0.018 OR 1.05 [1.01–1.09].

At multivariate analysis, all the previous parameters were statistically significant (except age ≥ 28 years old, *p* = 0.539 OR 1.09 [0.82–1.45]): male sex *p* = 0.001 OR 1.88 [0.41–2.51], familiarity for dyslipidemia *p* = 0.001 OR 2.82 [1.72–4.59], BMI ≥ 30 kg/m^2^ *p* = 0.001 OR 2.53 [1.46–4.38], and fat mass *p* = 0.001 OR 2.29 [1.38–3.80].

## 8. Lipid Athlete Score

According to the results of our study, we proposed the “Lipid Athlete Score” to identify athletes with abnormal lipid profiles, likely at high risk for future atherosclerotic disease.

We summarized the lipid athlete score in Table 4. Major criteria included the definition of dyslipidemia according to the cut-off of LDL (≥115 mg/dL) and the introduction of a new cut-off of the LDL/HDL ratio (≥1.90). The minor criteria were male sex, BMI > 30 kg/m^2^, fat mass > 22% for males and 32% for females, familiarity for dyslipidemia, and the presence of conventional CV risk factors (i.e., hypertension, smoke habit, and diabetes).

**Figure 1:**
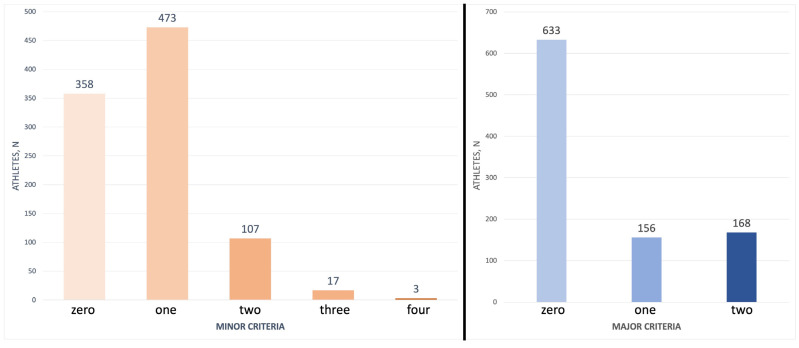
Athletes’ distribution according to the number of minor and major criteria of the lipid athlete score. Most of the athletes presented none or a few minor and major criteria of the lipid athlete score.

The distribution of athletes according to the number of minor (m) and major (M) criteria is shown in Figure 1.

By using the major and minor criteria, we proposed to classify the CV risk in our cohort as follows: high risk: 2 M + ≥ 3 m; medium risk: 2 M + 1–2 m or 1 M + ≥ 2 m; low risk: 2 M, 1 M + 1 m or ≥2 m; and no risk: 1 M or 1 m.

The athletes that were classified according to this criterion were distributed as follows: high-risk profiles were present in 12 athletes (1.2%), medium risk in 150 athletes (15.7%), low risk in 171 athletes (17.9%), and 624 (65.2%) were at no risk. In the small high-risk group, there were no endurance athletes, but four power, four skills, and four mixed ones.

## 9. Discussion

Congenital or acquired lipid metabolism disorders are major risk factors for atherosclerotic CV diseases [19]. It is estimated that one-third of the burden of ischemic heart disease worldwide refers to high cholesterol levels [20]. Cholesterol reduction is therefore the gold standard approach in CV preventive strategies [1].

It is known that the atherosclerotic process develops at a very young age and progresses silently for a long time. The onset already refers to the intrauterine period; thickening of the coronary intima in fetuses and infants has been shown [21], with further progression in children and adolescents [22]. Thickening of the intima represents the initial patho-morphological basis for lipid accumulation, resulting in atherosclerotic plaque formation.

Due to their regular physical activity, athletes are considered as low CV risk and are intuitively recognized to be a healthy lifestyle model. However, several studies have questioned this conviction by showing surprising high rates of CV risk factors in young and adult athletes, both active and retired [2,23,24,25].

At present, data on CV risk profiles from large cohorts of competitive athletes are relatively scarce, and these categories are still under-represented in large epidemiologic studies and clinical trials.

Moreover, it is well known that atherosclerotic disease, closely related to dyslipidemia, is responsible for the majority of adverse cardiac events in adult and senior athletes (over 35 years of age) [26,27]. In this regard, a remarkable point is that premature atherosclerosis leads to death in a surprisingly large group of competitive young athletes, accounting for 2–20% of the cases of sudden cardiac deaths, when it was long thought to be a cause of death almost exclusively in adult and senior athletes [28].

Furthermore, it has been pointed out that retired professional football players, despite their past as elite athletes, have comparable rates of subclinical atherosclerosis as a clinically referred population of obese and overweight men [29], and current data have advanced the hypothesis that long-term endurance exercise may speed up, rather than reduce, coronary atherosclerosis, being associated with a higher prevalence of calcific coronary plaques, through unknown and only speculative mechanisms [30,31,32,33].

Therefore, preventing the long-term evolution of subclinical atherosclerosis in a young, athletic population is essential to timely identifying dyslipidemia. At present, the ideal method for assessing the CV risk of young (<35 years old) competitive athletes remains controversial [27]. Indeed, CV risk assessment using the tools recommended by the ESC guidelines (SCORE and SCORE2 algorithms) are unsuitable, as they are aimed at individuals above 40 years old and do not consider physical fitness [34].

Moreover, athletes are a globally low-risk population who might benefit more from a comprehensive risk assessment rather than an analysis of risk based on lipid levels alone [35], and a lifetime benefit perspective of an early intervention may be useful despite the short-term CV risk potentially being low [36].

At present, no evidence-based targets or “scores” specific for young athletes are implemented in the clinical practice. Hence, in this study, we evaluated the distribution of anthropometrics, CV risk factors, and lipid profiles in a large cohort of Olympic athletes of both sexes and different sporting disciplines, aiming to develop a specific score (i.e., the lipid athlete score) that overcomes the limitations of individual parameters. This tool would allow to define subjects with dyslipidemia more accurately, and to define individuals at greater risk of atherosclerotic disease more precisely.

According to the lipid athlete score, from a total of 343 dyslipidemic athletes, defined by the LDL cut-off ≥115 mg/dL and the LDL/HDL ratio ≥ 1.90, only 12/343 had a high-risk profile.

It is worth highlighting that the great majority (83%) of our athlete population was at low or no risk, and that, remarkably, only a small subset of individuals (1.2%) showed a high-risk profile, among whom they were not engaged in endurance sports.

In our athlete population, we confirm that endurance sports showed the most favorable lipid profile, with a significant increase in HDL cholesterol, in association with better anthropometric parameters (overweight and increased fat mass were not represented in this subgroup) and fewer risk factors. Conversely, skill disciplines were associated with an increase in TG. Therefore, the sport category has a noticeable effect on the CV risk profile, in agreement with results from previous studies [2,37], highlighting the advantageous influences of aerobic exercise on CV health.

Furthermore, the number of female athletes participating in various sports at elite levels has grown in current years, and sex differences, related to physiological and particularly hormonal variations (higher circulating estrogen and lower androgen levels), have been shown to influence CV adaptations to exercise. In this regard, in our cohort, it is to be noted that women exhibited a lower CV risk profile (lower body weight, lower BMI, lower waist circumference, etc.), and a better lipid profile (higher HDL, lower LDL, and lower LDL/HDL ratio); these data add further elements in understanding the sex-related differences in the CV risk profile and the incidence of atherosclerotic disease.

It is also relevant to note that in our study, aging correlates with an increase in the BMI and an increase in the fat mass percentage, and these findings confirm that age, as expected, is the major driver of CVD risk, starting even from the younger ages.

At the same time, an increase in TC, LDL, and TG, together with a progressive reduction in HDL cholesterol and a consequently higher LDL/HDL ratio, correlated with an increase in body weight and fat mass. These results agree with data reported in previous studies and findings in the general population [38].

Overall, in spite of the protective effect of intense and routine exercise, athletes are not completely immune to CV risk factors, among which dyslipidemia constitutes one of the most prevalent. The lipid athlete score described here could allow for a more precise definition of the risk profile by placing such factors in a more comprehensive context by allowing an early prevention strategy to decrease the rates of cardiac events in athletes.

## 10. Limitations

Our study has several limitations. The main limitation of preventive studies trying to identify and early treat high-risk young populations is the lack of concrete CV events, so randomized or retrospective studies miss their strength for two practical reasons. First, since the event rates are much smaller in young individuals, the number of participants to be enrolled would be in the tens of thousands. Second, the study costs would be exorbitant. A 25–30-year prevention trial based on the future development of CV clinical events in a general population will likely never be realized, especially in selected populations, like elite athletes.

Most importantly, the irrefutable results of statin intervention trials, in the general population and in sub-populations that have been studied, leave no doubt that reducing bad cholesterol levels reduces overall CV risks; this hypothesis is strongly supported from epidemiologic correlations, pathologic observations, mechanistic studies, and animal model studies [39,40].

Moreover, our score has not yet been validated, and it represents an attempt to provide a theoretical tool that can improve the identification of dyslipidemic subjects among athletes and the classification of their CV risk.

Another limitation is represented by the demographic characteristics of the analyzed population: a small age range (most of them under 30 years old), a large sample, but still limited to a single center; it includes mainly Caucasians and athletes of exclusively Italian nationality, limiting generalizability.

Lastly, a further limitation is the retrospective observational design of the present study with the lack of outcome data. Hence, upcoming prospective studies are required to validate the proposed score and to evaluate its prognostic impact on CV health.

## 11. Future Perspectives

In the future, we seek to implement the newly defined Athlete’s lipid score in larger cohorts of individuals, comprising a broad range of ages and levels of training and achievement, to validate the feasibility of the score. Indeed, prospective longitudinal observational studies are needed to assess the clinical value of the score in the management of lipid disorders in athletic populations.

## 12. Conclusions

The present study highlights that regular exercise training, although constituting a well-known cardioprotective factor, does not eliminate the occurrence of risk factors in world-class athletes. Dyslipidemia was frequent in elite athletes and affected a considerable part of our population (35.8%), in accordance with optimized cut-offs for athletes. At present, specific targets or “scores” for athletes are lacking. Therefore, we have defined a new clinical tool, named the lipid athlete score, based on simple lipid parameters and derived score indexes for the better stratification of “high-risk” subjects. In accordance with this tool, a substantial proportion of Olympic athletes were at no or low risk (83%), and only a minor percentage (1.2%) presented a high CV risk profile.

In conclusion, our study emphasizes the importance of accurately identifying and contextualizing, even at a young age and in high-caliber athletes, modifiable CV risk factors, with a focus on abnormal lipid profiles, to start the appropriate prevention strategies.

## Figures and Tables

**Table 1:** Anthropometrics, cardiovascular risk factors, and lipid profiles of Olympic athletes according to different sport categories.

	Power	Skills	Endurance	Mixed	*p*-Value (Pooled)	*p*-Value (Pairwise Analysis)
n (%)	311 (32.5)	122 (12.8)	264 (27.6)	260 (27.1)		
Age, years	25.9 ± 4.6	28.6 ± 6.5	27.2 ± 4.5	27.6 ± 4.7	0.001	“Power” significantly younger (*p* < 0.001 in all pairwise comparisons; other comparison with *p* > 0.05)
Male, n (%)	173 (55.6)	71 (58.1)	161 (60.9)	125 (48)	0.025	“Mixed” lower incidence of male sex (*p* = 0.001 in all comparisons)
Black, n (%)	18 (5.8)	0 (0)	10 (3.8)	6 (2.3)	0.017	-
Smokers, n (%)	12 (3.8)	16 (13.1)	1 (0.4)	48 (18.4)	0.001	“Endurance” lower incidence of smokers (all *p* = 0.001); “Mixed” higher incidence of smokers (all *p* = 0.001)
Familiarity for ATH, n (%)	69 (22.1)	36 (29.5)	53 (20)	67 (25.7)	0.157	-
Familiarity for dyslipidemia, n (%)	20 (6.43)	13 (10.6)	21 (7.9)	24 (9.2)	0.445	-
Weight, kg	73.8 ± 15.5	72.1 ± 14.1	69.6 ± 13.7	76.6 ± 16.3	0.001	“Endurance” significantly less weight (*p* < 0.001 in all pairwise comparisons; other comparison with *p* > 0.05)
BMI, kg/m^2^	23.3 3.6	23.9 ± 3.3	22.0 ± 2.6	23.4 ± 2.8	0.001	“Endurance” significantly less BMI (*p* < 0.001 in all pairwise comparisons; other comparison with *p* > 0.05)
Fat mass, %	15 ± 6.3	19.7 ± 7.4	13.3 ± 5.3	17.4 ± 6.4	0.001	All pairwise comparisons have statistically significant differences
Waist, cm	75.9 ± 8.7	75.5 ± 7.7	74.4 ± 7.5	77.9 ± 8.4	0.098	-
W/H ratio	0.78 ± 0.05	0.79 ± 0.06	0.78 ± 0.05	0.78 ± 0.06	0.541	-
Kcal	2893 ± 681	2231 ± 482	2811 ± 733	2508 ± 717	0.001	Power vs. endurance, *p* = 0.353 “Power” and “Endurance” statistically significant differences with other groups (all *p* = 0.001)
Fat in diet, %	29.3 ± 3.7	29.8 ± 6.5	29.2 ± 3.3	29.7 ± 3.2	0.231	-
Alcohol, n (%)	179 (57.5)	67 (55)	149 (56.4)	160 (61.5)	0.552	-
TC, mg/dL	175.6 ± 33.1	175.3 ± 34.7	180.3 ± 31.4	182.5 ± 32.3	0.075	-
LDL, mg/dL	97.8 ± 27.1	97.7 ± 29.2	95.1 ± 26.9	102 ± 28.1	0.100	-
HDL, mg/dL	62.8 ± 14.9	61 ± 16.5	71.6 ± 16.3	66.4 ± 14.6	0.001	“Endurance” higher values (all comparison *p* = 0.001); no significant differences among other groups
TG, mg/dL	75.7 ± 40.5	85.1 ± 56.9	68.6 ± 28.7	73.8 ± 36.4	0.009	“Skills” higher values (all comparison *p* = 0.001); no significant differences among other groups
LDL/HDL	1.6 ± 0.6	1.7 ± 0.7	1.4 ± 0.5	1.6 ± 0.6	0.264	-

Endurance athletes presented the lowest global cardiovascular risk compared to other types of sports, with a better lipid profile characterized by higher HDL-cholesterol and the lowest TG level compared to other sporting disciplines. Higher levels of TG were observed in athletes practicing more “sedentary” sports (skills). Abbreviations: ATH: atherosclerosis; BMI: body mass index; HDL: high-density lipoprotein; LDL: low-density lipoprotein; TC: total cholesterol; TG: triglycerides; and W/H: waist/hip.

**Table 2:** Gender differences in anthropometrics, cardiovascular risk factors, and lipid profiles.

	Total		*p*-Value
	Male	Female	
n (%)	530 (55.4)	427 (44.6)	
Age, years	27.6 ± 5	26.4 ± 4.8	0.005
Black, n (%)	22 (4.1)	12 (2.8)	0.265
Smokers, n (%)	46 (8.7)	31 (7.3)	0.422
Familiarity for ATH, n (%)	125 (23.5)	100 (23.4)	0.952
Familiarity for dyslipidemia, n (%)	42 (7.9)	36 (8.4)	0.776
Weight, kg	81.6 ± 13.5	62.7 ± 10	0.001
BMI, kg/m^2^	24.2 ± 3.2	21.7 ± 2.6	0.001
Fat mass, %	12.1 ± 3.2	20.2 ± 5.4	0.001
Waist, cm	81.4 ± 7	70 ± 4.8	0.001
W/H ratio	0.82 ± 0.04	0.74 ± 0.04	0.001
Kcal	2951 ± 706	2271 ± 519	0.001
Alcohol	326 (61.5)	229 (53.6)	0.014
Fat, %	29.6 ± 3.8	29.3 ± 4.2	0.245
TC, mg/dL	177 ± 32.5	181 ± 32.9	0.099
LDL, mg/dL	100.8 ± 29.4	95 ± 25.1	0.002
HDL, mg/dL	60.7 ± 14.8	72.4 ± 14.9	0.001
TG, mg/dL	79.5 ± 46.6	68.2 ± 27.1	0.001
LDL/HDL	1.76 ± 0.7	1.36 ± 0.5	0.001

Female athletes presented concrete differences in anthropometric parameters (characterized by lower body weight, BMI, and waist circumference and higher fat mass) and a better lipid profile compared to men (lower LDL, TG, and LDL/HDL, with higher HDL cholesterol). Abbreviations: ATH: atherosclerosis; BMI: body mass index; HDL: high-density lipoprotein; LDL: low-density lipoprotein; TC: total cholesterol; TG: triglycerides; and W/H: waist/hip.

**Table 3:** Anthropometrics, sport category, cardiovascular risk factors, and lipid profile differences according to BMI and fat mass % in males and females.

	BMI					Fat Mass					
	<25	25–29.9	30–34.9	≥35	*p*-Value	Male		*p*-Value	Female		*p*-Value
						≤22	>22		≤32	>32	
n (%)	739 (77.2)	192 (20)	19 (2)	7 (0.7)		504 (95.1)	26 (4.9)		417 (96.7)	10 (2.3)	
Age, years	26.6 ± 4.8	28.6 ± 4.8	31.5 ± 5.5	32.1 ± 5.9	0.001	27.3 ± 4.8	33.8 ± 6	0.001	26.4 ± 4.7	30.1 ± 5.9	0.015
Male, n (%)	348 (47.1)	160 (83.3)	17 (89.5)	5 (71.4)	0.001	504 (100)	26 (100)	-	0 (0)	0 (0)	-
Black, n (%)	28 (3.8)	5 (2.6)	1 (5.3)	0 (0)	0.790	25 (5)	0 (0)	0.277	12 (2.9)	0 (0)	0.586
Smokers, n (%)	60 (9.5)	15 (7.8)	1 (5.3)	1 (14.2)	0.899	71 (14.1)	2 (7.7)	0.855	31 (7.4)	0 (0)	0.371
Familiarity for ATH, n (%)	174 (23.5)	45 (23.4)	5 (26.3)	1 (14.2)	0.937	166 (32.9)	5 (19.2)	0.592	97 (23.3)	3 (30)	0.619
Familiarity for dyslipidemia, n (%)	65 (8.8)	10 (5.2)	2 (10.6)	1 (14.2)	0.371	59 (11.7)	4 (15.4)	0.149	36 (8.6)	0 (0)	0.332
Power, n (%)	226 (30.6)	73 (38)	7 (36.8)	5 (71.4)	0.039	164 (32.5)	9 (34.6)	0.826	135 (32.4)	3 (30)	0.874
Skills, n (%)	87 (11.8)	28 (14.6)	6 (31.6)	1 (14.2)	0.064	59 (11.7)	12 (46.1)	0.001	47 (11.3)	4 (40)	0.006
Endurance, n (%)	231 (31.2)	33 (17.2)	0 (0)	0 (0)	0.001	161 (31.9)	0 (0)	0.001	103 (24.7)	0	0.071
Mixed, n (%)	194 (26.2)	59 (30.7)	6 (31.6)	1 (14.2)	0.514	120 (23.8)	5 (19.2)	0.592	132 (31.7)	3 (30)	0.911
Weight, kg	68.1 ± 11.9	87.8 ± 10.4	102.7 ± 9.4	125.3 ± 12.9	0.001	80.8 ± 12.4	98.5 ± 20.8	0.001	62.1 ± 8.9	87.1 ± 17.9	0.001
BMI, kg/m^2^	21.8 ± 1.9	26.6 ± 1.2	31.6 ± 1.5	37.2 ± 2.1	0.001	24 ± 2.7	28.9 ± 6.8	0.001	21.5 ± 2.2	29.9 ± 4.2	0.001
Fat mass, %	15.4 ± 6.2	16.1 ± 6.6	22.6 ± 9.8	29.7 ± 5.9	0.001	11.4 ± 4	26.2 ± 3.5	0.001	19.9 ± 4.8	36.5 ± 2.8	0.001
Waist, cm	73 ± 6.3	84.4 ± 6	92.3 ± 5.9	118 ± 3.4	0.001	81.1 ± 6.3	95.1 ± 16.2	0.001	69.8 ± 4.6	84.5 ± 2.5	0.001
W/H ratio	0.78 ± 0.05	0.82 ± 0.05	0.85 ± 0.05	0.93 ± 0.04	0.001	0.82 ± 0.04	0.88 ± 0.07	0.001	0.74 ± 0.04	0.8 ± 0.07	0.001
TC, mg/dL	178.9 ± 33.1	177.1 ± 31.2	186.8 ± 34.5	193.1 ± 23.1	0.353	176.2 ± 32.1	197.1 ± 33.7	0.001	180.5 ± 33.1	192.1 ± 42.1	0.272
LDL, mg/dL	96.9 ± 27.1	100.7 ± 28.3	114 ± 33.6	126.5 ± 27.3	0.001	99.6 ± 28.7	124.5 ± 31.8	0.001	94.7 ± 25.3	107.1 ± 23.5	0.126
HDL, mg/dL	68.2 ± 15.4	59.5 ± 15.5	52.6 ± 12.1	46.2 ± 4.8	0.001	61.2 ± 14.9	51.4 ± 10.8	0.001	72.6 ± 14.9	63.5 ± 14.6	0.056
TG, mg/dL	70.5 ± 31.4	85.5 ± 56.8	104.8 ± 62.5	103.5 ± 34.8	0.001	78.2 ± 46.6	106.9 ± 56.2	0.002	67.2 ± 24.9	110.8 ± 58.8	0.001
LDL/HDL	1.50 ± 0.5	1.8 ± 0.7	2.32 ± 0.9	2.81 ± 0.8	0.001	1.72 ± 0.6	2.51 ± 0.8	0.001	1.35 ± 0.5	1.74 ± 0.5	0.008

Lipid parameters were directly correlated to anthropometric parameters. With the increase in BMI and fat mass, increases in TC, LDL, and TG were observed, with a progressive reduction in HDL cholesterol. Abbreviations: ATH: atherosclerosis; BMI: body mass index; HDL: high-density lipoprotein; LDL: low-density lipoprotein; TC: total cholesterol; TG: triglycerides; and W/H: waist/hip.

**Table 4:** Athletes’ lipid score.

	Variable
Major criteria (M)	LDL ≥ 115 mg/dL
	LDL/HDL ratio ≥ 1.90
Minor criteria (m)	Male sex
	BMI > 30 or fat mass > 22% for males and 32% for females
	Familiarity for dyslipidemia
	Cardiovascular risk factors (smoke, hypertension, and diabetes)

Protective factors: endurance sports. High risk: 2 M + ≥ 3 m; medium risk: 2 M + 1–2 m or 1 M + ≥ 2 m; low risk: 2 M or 1 M + 1 m or ≥2 m; and no risk: 1 M or 1 m.

## Data Availability

The data presented in this study are available on request from the corresponding author.
